# Sedative effect of remimazolam combined with alfentanil in colonoscopic polypectomy: a prospective, randomized, controlled clinical trial

**DOI:** 10.1186/s12871-022-01805-3

**Published:** 2022-08-16

**Authors:** Yueyang Xin, Tiantian Chu, Jinxu Wang, Aijun Xu

**Affiliations:** Department of Anesthesiology, Tongji Hospital, Tongji Medical College, Huazhong University of Science and Technology, 1095 Jiefang Avenue, Wuhan, 430030 China

**Keywords:** Alfentanil, Colonoscopic surgical procedure, Deep sedation, Remimazolam besylate

## Abstract

**Background:**

Remimazolam is a newer benzodiazepine with properties of rapid onset, short duration of action, and fast recovery. Our study was to evaluate the effects of different doses of remimazolam combined with alfentanil in colonoscopic polypectomy.

**Methods:**

One hundred twenty patients were randomly divided into four groups: alfentanil and propofol (AP) group, alfentanil and remimazolam 0.1 mg/kg (AR1 group), 0.15 mg/kg (AR2 group), or 0.2 mg/kg (AR3 group). Patients in the four groups received alfentanil 10 μg/kg, followed by propofol 2 mg/kg and three dosages of remimazolam. Modified Observer's Assessment of Alertness and Sedation (MOAA/S) scale, heart rate (HR), oxygen saturation (SpO_2_), respiratory rate (RR), bispectral index (BIS) values and mean arterial pressure (MAP) were collected at intervals of 5 min and analyzed at different time points: before anesthesia (T0), 5 min (T1), 10 min (T2), 15 min after anesthesia (T3) and at the end of surgery (T4). The average MAP was calculated utilizing the average of all MAP values. The primary outcome was the success rate of sedation. Secondary outcomes included time to full alert and adverse events.

**Results:**

The success rate of sedation was 100% among the four groups. The incidence of hypotension was significantly decreased (all *P* < 0.05) and the average MAP was higher in AR1-AR3 groups than AP group (all *P* < 0.001). None of the patients developed bradycardia or hypertension during surgery in all study groups. BIS values were higher (all *P* < 0.001) and the time to full alert was statistically shorter in AR1-AR3 groups (all *P* < 0.05) compared with the AP group. The MOAA/S score in AR1 was higher than AR2 (*P* < 0.05) and the AR3 group (*P* < 0.05) at T1 and BIS values in the AR1 group were significantly higher than AR3 group (*P* < 0.05) at T4.

**Conclusions:**

Remimazolam combined with alfentanil have a non-inferior sedative effect than propofol during the colonoscopic polypectomy. Moreover, this combination of two short-acting drugs might be a safer alternative.

**Trial registration:**

The clinical trial was registered on (16/05/2021, ChiCTR2100046492).

**Supplementary Information:**

The online version contains supplementary material available at 10.1186/s12871-022-01805-3.

## Background

During colonoscopic polypectomy, a minimally invasive surgical procedure of colonoscopy requires continuous inflation of the abdominal wall to clear the field of vision. Stimulation and traction of the intestinal wall cause excitation of the visceral nerve, resulting in bradycardia, body movement, patient discomfort, and interference with surgery [1]. For procedures like colonoscopic polypectomy requiring minimally invasive surgical interventions, anesthesiologists need to provide adequate sedoanalgesia to provide optimal surgical conditions for 20 to 30 min without respiratory depression desired for the comfort of both the patient and the surgeon, which we call monitored anesthesia care (MAC), in order to prevent complications [2–5]. However, to keep the airway patent and maintain stable hemodynamics, excessive depth of sedation is also not recommended [5].

Propofol has the advantages of rapid onset and quick recovery and is often used in gastroscopy [6]. However, propofol quickly causes adverse side effects, including hypotension and hypoxemia [7–9], and propofol injection pain also cannot be ignored [10]. Alfentanil is a short-acting opioid analgesic with high analgesic intensity and similar complication risks to other opioids. A shorter recovery time makes alfentanil meeting the needs of daytime anesthesia more suitable [11–14].

Remimazolam is a novel short-acting intravenous benzodiazepine that acts as a positive allosteric modulator of the γ-aminobutyric acid subtype A (GABA_A_) receptor via the benzodiazepine binding site [15, 16]. It retains the characteristics of benzodiazepines, such as water solubility, antagonism, and no injection pain. It also possesses some pharmacological characteristics of remifentanil due to the methyl propionate side chain in the structure, such as rapid onset, short duration of action, inactivity of metabolites and not being affected by infusion time [17–19]. The success rate of sedation of remimazolam was reported to be non-inferior to propofol and provided a lower incidence of adverse events [20, 21]. Nevertheless, there is still no clear conclusion about using alfentanil combined with remimazolam in colonoscopic polypectomy. Therefore, our study aims to evaluate three different dosages of remimazolam combined with alfentanil for sedation induction in patients undergoing colonoscopic polypectomy.

## Methods

### Study design and setting

This prospective, randomized, controlled pilot trial was designed to observe patients undergoing colonoscopic polypectomy in Tongji Hospital. Tongji Medical College of Huazhong University of Science and Technology Ethics Committee (IORG No: IORG0003571) approved the conduct of the trial. The trial was registered before patient enrollment at http://www.chictr.org.cn (principal investigator: Aijun Xu, date of registration and registration number: 16/05/2021, ChiCTR2100046492) and written informed consent was obtained from all subjects participating in the study. This study follows applicable Consolidated Standards of Reporting Trials (CONSORT) guidelines.

### Participants and recruitment

Eligible patients who underwent colonoscopic polypectomy in Tongji Hospital from May 2021 to March 2022 were evaluated according to the inclusion criteria: 1) aged 18 to 80 years; 2) American Society of Anesthesiologists' (ASA) status I or II; 3) operation time is 20 to 60 min; 4) Body Mass Index (BMI) 18.5 to 23.9 kg/m^2^. The exclusion criteria are as follows: 1) emergency operation; 2) with a high risk of a full stomach and reflux aspiration; 3) allergic to benzodiazepines and opioids; 4) take sedative, analgesic, or antidepressant drugs within 24 h; 5) pregnant or breastfeeding; 6) with abnormal liver and kidney function; 7) with a history of drug abuse; 8) recently participated in other clinical studies; 9) Patients who cannot cooperate with communication. The dropout criteria are as follows: 1) severe adverse events such as massive hemorrhage and intestinal perforation during the operation; 2) participant withdrew informed consent; 3) investigators determined that the patient withdrew if a person had poor compliance, serious complications such as postoperative intestinal perforation in need of emergency surgery and severe infection. Detailed reasons will be recorded, and the case report form (CRF) will be retained for reference.

### Randomization and blinding

Randomization was implemented by researchers who were not involved in anesthesia management and intraoperative and postoperative follow-up to avoid selection bias. Patients were randomized into four groups according to the randomized number lists generated by Statistical Package for Social Sciences (SPSS) software version 26.0. Randomized numbers were sealed in numbered opaque envelopes according to which patients were included.

In this study, a single-blind study method was adopted. An investigator was assigned to determine the order of patients and coordinate the relationship between researchers. Another investigator, blinded to the study protocol and trained on evaluation methods before the study, performed the preoperative evaluation and postoperative follow-up. An anesthesiologist performed anesthesia management and intraoperative data collection. The statistical experts of Tongji Hospital analyzed the final data. All researchers except anesthesiologists were blinded to the grouping.

### Anesthesia management and intervention

**Fig. 1:**
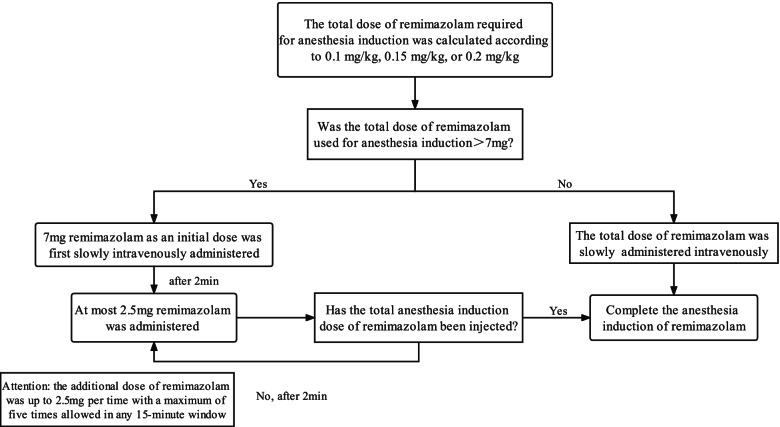
The administration of remimazolam at sedation induction

Patients who underwent bowel preparation were established venous access and introduced 250 mL 0.9% sodium chloride solution when brought to the endoscopy room. Blood pressure (BP), heart rate (HR), oxygen saturation (SpO_2_), respiratory rate (RR) and bispectral index (BIS) were routinely monitored. Following lateral positioning, an oxygen inhalation mask was administered immediately at a rate of 3L/min. The patients in the alfentanil and propofol (AP) group were administered alfentanil (Yichang Humanwell Pharmaceutical, Co., Ltd., China, 13S03051) 10 μg/kg and propofol (Corden Pharma S.P.A., RX061) 2 mg/kg [22–25]. Patients in remimazolam groups were received alfentanil 10 μg/kg, followed by remimazolam besylate (Yichang Humanwell Pharmaceutical, Co., Ltd., China, 70,705,021) 0.1 mg/kg (AR1 group), 0.15 mg/kg (AR2 group), or 0.2 mg/kg (AR3 group). It took over 1 min to induce sedation for all patients. When the Modified Observers Assessment of Alertness and Sedation (MOAA/S) score ≤ 1 [24], colonoscopy was performed by the same endoscopist who had over ten years of experience. Remimazolam was administered strictly following the instruction, as shown in Fig. 1. Additional 1/3 to 1/2 of the initial dose of alfentanil or propofol and 2.5 mg remimazolam were administered to keep the appropriate sedation and painless according to the surgery duration. If the initial and supplemental boluses of remimazolam reached the maximum dose according to instruction within a 15-min window, 0.5-1 mg/kg propofol was administered as rescue sedative medication when requested to maintain enough sedation (MOAA/S score ≤ 1). Artificial assisted ventilation was given immediately if a decrease in SpO_2_ to less than 90% and sustained for more than 20 s, which was regarded as respiratory depression associated with sedation [26]. If obvious hypotension (20% lower than base value) and bradycardia (HR is less than 60 beats per minute) occur, ephedrine and atropine were given to maintain circulation stability.

### Postoperative management

After surgery, patients were monitored in the post anesthesia care unit (PACU). After meeting the PACU standards (Steward score > 4), patients were transferred to the ward. Follow-up was performed to evaluate postoperative adverse reactions 24 h after the surgery, including hypotension, hypertension, bradycardia, tachycardia, pain, vertigo, nausea, vomiting, and intraoperative awareness.

### Outcomes and data collection

Baseline demographics and case characteristics were recorded, such as age, sex, height, and weight. The primary outcome is the success rate of sedation. Successful sedation for the colonoscopic polypectomy was defined based on a previous study [21] as follows: (1) completion of the procedure, (2) no requirement for a rescue sedative, (3) administration up to a maximum of five supplemental doses within 15 min of the initial dose.

The secondary outcomes were (I) time of anesthesia; (II) time of operation; (III) time to full alert; (IV) duration of PACU; (V) dose of drugs used for anesthesia management; (VI) intraoperative adverse reactions such as body movement, cough, hypotension, hypertension, bradycardia, tachycardia, (VII) postoperative adverse reactions including hypotension, hypertension, bradycardia, tachycardia, pain, vertigo, nausea, vomiting, intraoperative awareness.

MOAA/S scale (Table 1) was recorded to determine the level of sedation every five minutes from anesthetic administration until the end of the procedure. MAP, HR, SpO_2_, BIS and RR were recorded every five minutes. The average MAP was calculated utilizing the average of all MAP values collected at intervals of 5 min during the operation [27].

**Table 1:** Modified Observer's Assessment of Alertness and Sedation (MOAA/S) Scale

Score	Responsiveness
5	Subject responds readily to name spoken in a normal tone
4	Lethargic response of a subject to a name spoken in a normal tone
3	The subject responds only after a name is called loudly and repeatedly
2	The subject responds only after mild prodding or shaking
1	The subject responds only after a painful trapezius squeeze
0	The subject does not respond to painful trapezius squeeze

### Sample size

Prior studies suggested the success rate of sedation was 96.52% to 97.34% for remimazolam and 100% for propofol applied in colonoscopy [20, 21, 28]. In our trial, propofol combined with alfentanil served as an active control. A non-inferiority test was put into effect on the four groups' primary outcome (the success rate of sedation). The predefined non-inferiority margin was 8%. The sample size was calculated using Power Analysis and Sample Size (PASS) 15.0.5 software based on the following parameters: the success rate of sedation was 96.93% (the median of 96.52% to 97.34%) of remimazolam and 99.99% of propofol, the ratio of remimazolam groups (0.1 mg/kg, 0.15 mg/kg and 0.2 mg/kg) to propofol group was 3:1. A sample size of 26 per group was calculated using two-sided type I error rate of 5% (α = 0.05) and 80% power (β = 0.1). We estimated that 15% dropped out of the study; thus, 30 cases for each group had to be included. Finally, 120 patients were included as the sample size in this study.

### Statistical analysis

The Shapiro–Wilk test was used to determine the normal distribution of continuous variables. According to the distribution, the mean ± standard deviation (SD) or median (interquartile range) were used to describe continuous variables. Numbers (percentage) were used to describe categorical data (such as gender and MOAA/S scores). Continuous variables were analyzed using one-way analysis of variance (ANOVA) or Welch ANOVA based on the homogeneity of variance test and Kruskal-Walli's test and then followed by Bonferroni's post hoc test or Games-Howell's post hoc test to compare differences among groups. Categorical variables were compared using Pearson's chi-square test or Fisher's exact test, followed by Bonferroni's post hoc test. *P* value < 0.05 was considered statistically significant. Date analyses were generated by SPSS software version 26.0 (SPSS Inc., Chicago, IL, USA) and GraphPad Prism 8 (GraphPad Software, San Diego, CA, USA).

## Results

**Fig. 2:**
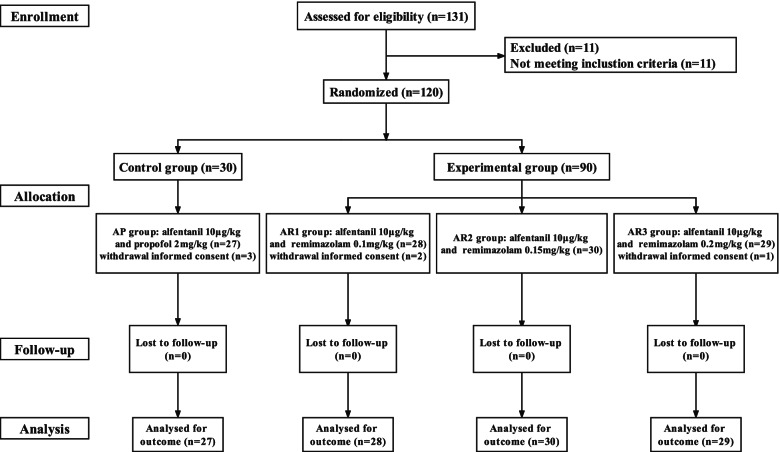
CONSORT flow diagram of participants

One hundred thirty-one patients were assessed for eligibility and 11 were excluded because of age, BMI and severe existing physical illnesses such as renal failure, heart failure, and cerebral infarction. 120 patients were randomized into four groups (n = 30 for each group). Protocol deviations included the withdrawal of informed consent (*n* = 6, Fig. 2). The baseline demographic of all groups was presented in Table 2.

**Table 2:** Patient demographics

Characteristics	AP Group (*n* = 27)	AR1 Group (*n* = 28)	AR2 Group (*n* = 30)	AR3 Group (*n* = 29)
age years	56.00 ± 10.13	54.11 ± 12.08	49.70 ± 8.95	52.24 ± 9.80
male/female	17/10	20/8	23/7	19/10
BMI, kg/m^2^	22.28 ± 1.61	22.20 ± 1.71	22.43 ± 1.77	22.40 ± 1.67
ASA classification				
I	20 (74.1%)	23 (82.1%)	24 (80.0%)	23 (79.3%)
II	7 (25.9%)	5 (17.9%)	6 (20.0%)	6 (20.7%)

*Note*: Data are presented as mean ± SD and number (percentage)

*Abbreviations*: *AP* Alfentanil and propofol, *AR1* Alfentanil and remimazolam (0.1 mg/kg), *AR2* Alfentanil and remimazolam (0.15 mg/kg), *AR3* Alfentanil and remimazolam (0.2 mg/kg), *ASA* American Society of Anesthesiologists physiological status, *BMI* Body mass index

### Primary outcome

The success rate of sedation during the colonoscopic polypectomy was 100% among the four groups (Table 3).

**Table 3:** The success rate of sedation

	AP Group (*n* = 27)	AR1 Group (*n* = 28)	AR2 Group (*n* = 30)	AR3 Group (*n* = 29)
Sedation success	27 (100%)	28 (100%)	30 (100%)	29 (100%)

*Note*: Data are presented as number (percentage)

*Abbreviations*: *AP* Alfentanil and propofol, *AR1* Alfentanil and remimazolam (0.1 mg/kg), *AR2* Alfentanil and remimazolam (0.15 mg/kg), *AR3* Alfentanil and remimazolam (0.2 mg/kg)

### Secondary outcomes

The secondary outcomes among the four groups were summarized in Table 4. There were no meaningful differences in the time of anesthesia (*P* = 0.794) and operation (*P* = 0.823) among all the groups. The time to full alert and PACU duration were significantly shorter in AR1—AR3 group ( all *P* < 0.05) than that in AP group. However, no significant difference was shown among three remimazolam groups.

**Table 4:** Secondary outcomes

Secondary outcomes	AP Group (*n* = 27)	AR1 Group (*n* = 28)	AR2 Group (*n* = 30)	AR3 Group (*n* = 29)	*P* value
time of anesthesia, min	31.41 ± 9.61	32.46 ± 8.52	33.50 ± 10.33	33.66 ± 9.08	*P* = 0.794
time of operation, min	29.52 ± 9.71	30.39 ± 8.48	31.43 ± 10.29	31.62 ± 9.02	*P* = 0.823
time to full alert, min	7.44 ± 2.38	4.75 ± 1.08^a^	4.63 ± 1.35^a^	5.31 ± 1.54^a^	*P* < 0.001
duration of PACU, min	5.37 ± 1.80	4.04 ± 1.48^a^	3.40 ± 0.72^a^	3.59 ± 0.87^a^	*P* < 0.001
type and dose of drugs used for intraoperative anesthesia management					
alfentanil, μg	772.22 ± 151.49	805.36 ± 181.22	788.33 ± 125.33	786.21 ± 136.21	*P* = 0.877
remimazolam, mg	NA	18.63 ± 3.68	19.75 ± 4.60	20.83 ± 5.33	*P* = 0.201
Propofol, mg	221.11 ± 55.91	NA	NA	NA	NA
intraoperative adverse reactions					
body movement	5 (18.5%)	7 (25.0%)	2 (6.6%)	4 (13.8%)	*P* = 0.052
cough	0	1 (3.6%)	0	0	*P* = 0.482
hypotension	24 (88.9%)	8 (28.6%)^a^	11 (36.7%)^a^	10 (34.5%)^a^	*P* < 0.001
bradycardia	7 (25.9%)	3 (10.7%)	1 (3.3%)	2 (6.9%)	*P* = 0.061
postoperative adverse reactions					
pain	2 (7.4%)	1 (3.5%)	0	1 (3.4%)	*P* = 0.372
nausea and vomiting	0	1 (3.5%)	0	1 (3.4%)	*P* = 0.735

*Note*: Data are presented as the mean ± SD and number (percentage)

*Abbreviations*: *AP* Alfentanil and propofol, *AR1* Alfentanil followed by remimazolam (0.1 mg/kg), *AR2* Alfentanil followed by remimazolam (0.15 mg/kg), *AR3* Alfentanil followed by remimazolam (0.2 mg/kg), *NA* Not applicable, *PACU* Post anesthesia care unit

^a^ *P* < 0.05 vs. AP group

**Fig. 3:**
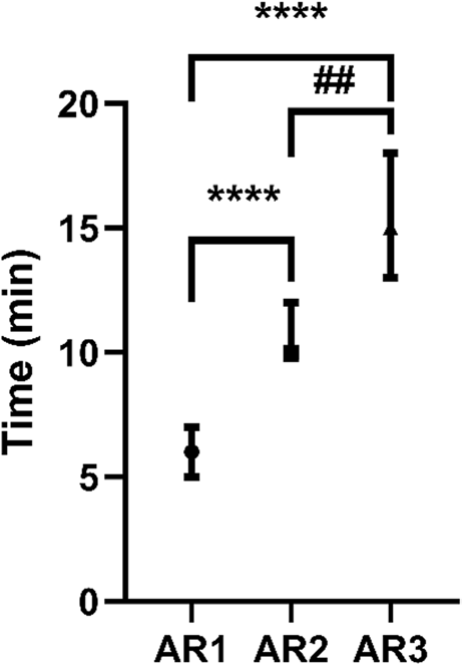
The time interval for the first addition of remimazolam. Compared with AR1 group, ^****^P < 0.0001, compared with AR2 group, ^##^*P* < 0.01. The time interval was presented as median (25th–75th centiles). Abbreviations: AR1 alfentanil and remimazolam (0.1 mg/kg); AR2 alfentanil and remimazolam (0.15 mg/kg); AR3 alfentanil and remimazolam (0.2 mg/kg)

After being injected with the dose of remimazolam for sedation induction, The median time interval for the first addition of remimazolam was 6 min in the AR1 group, 10 min in the AR2 group, and 15 min in the AR3 group. The time interval was significantly extended in the AR2 group (*P* < 0.0001) and AR3 group (*P* < 0.0001) compared with the AR1 group (Fig. 3). Moreover, the time interval was also significantly extended in the AR3 group (*P* < 0.01) compared with the AR2 group (Fig. 3). Alfentanil was added every 15 min after sedation induction for all study groups. There was no statistical difference between the doses of Alfentanil (*P* = 0.877) and remimazolam (*P* = 0.201) among AR1-AR3 groups for sedoanalgesia.

Though patients in AR2 and AR3 group had lower body movement rate than AR1 group, there was no significant difference among all groups (*P* = 0.052). Cough (*P* = 0.482) and bradycardia (*P* = 0.061) also differed insignificantly among all groups. The incidence of hypotension was significantly decreased for each dose of remimazolam (all *P* < 0.05), respectively, compared with propofol. Similarly, there was no significant difference among the remimazolam groups. None of the patients developed bradycardia or hypertension during surgery in all study groups.

Patients were followed up 24 h after surgery. Pain occurred in four patients and nausea and vomiting occurred in two patients. Appropriate treatment measures were given to alleviate the postoperative adverse reactions in the ward. There was no significant difference among all groups for pain (*P* = 0.372) and nausea and vomiting (*P* = 0.375). No patient developed other adverse reactions, including hypotension, hypertension, bradycardia, tachycardia, vertigo and intraoperative awareness.

### Outcomes of monitoring data

**Fig. 4:**
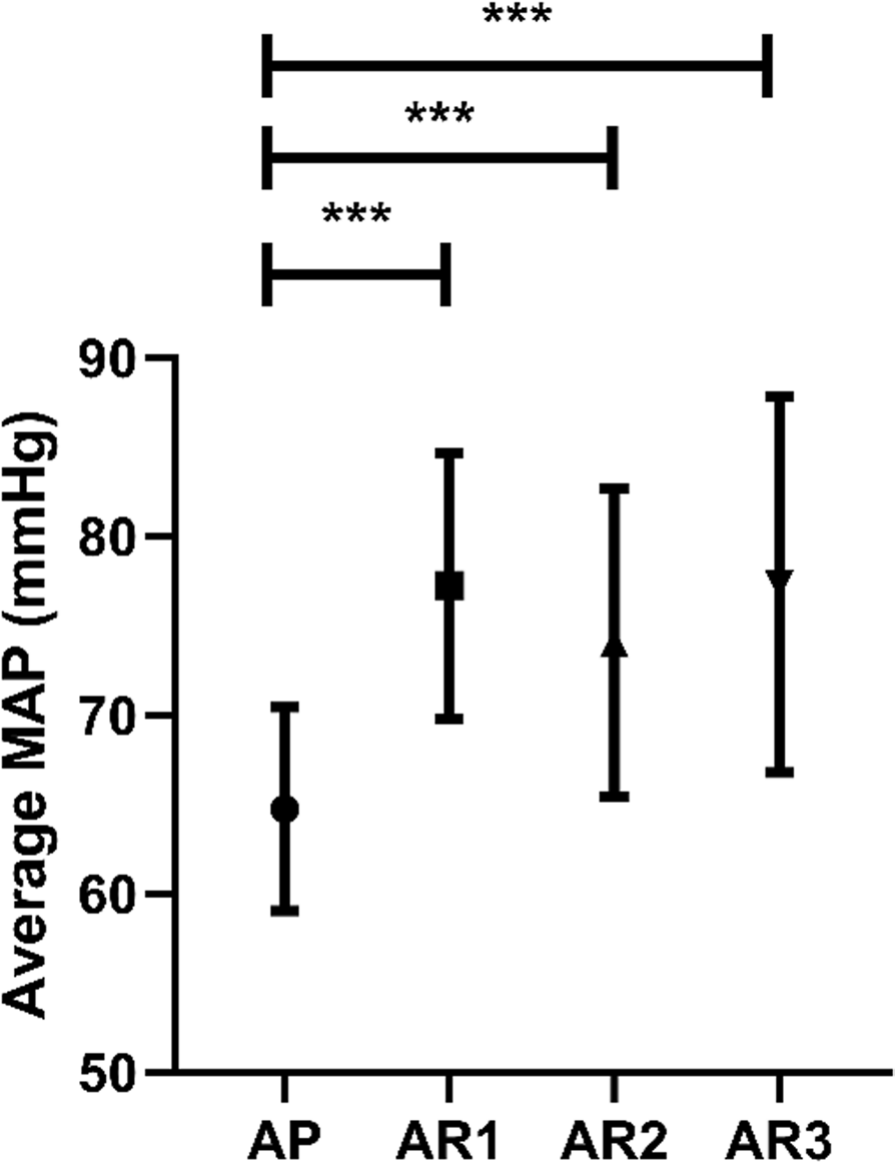
Average MAP. Compared with AP group, ***P < 0.001. The average MAP was presented as mean ± SD. Abbreviations: AP alfentanil and propofol; AR1 alfentanil and remimazolam (0.1 mg/kg); AR2 alfentanil and remimazolam (0.15 mg/kg); AR3 alfentanil and remimazolam (0.2 mg/kg)

**Fig. 5:**
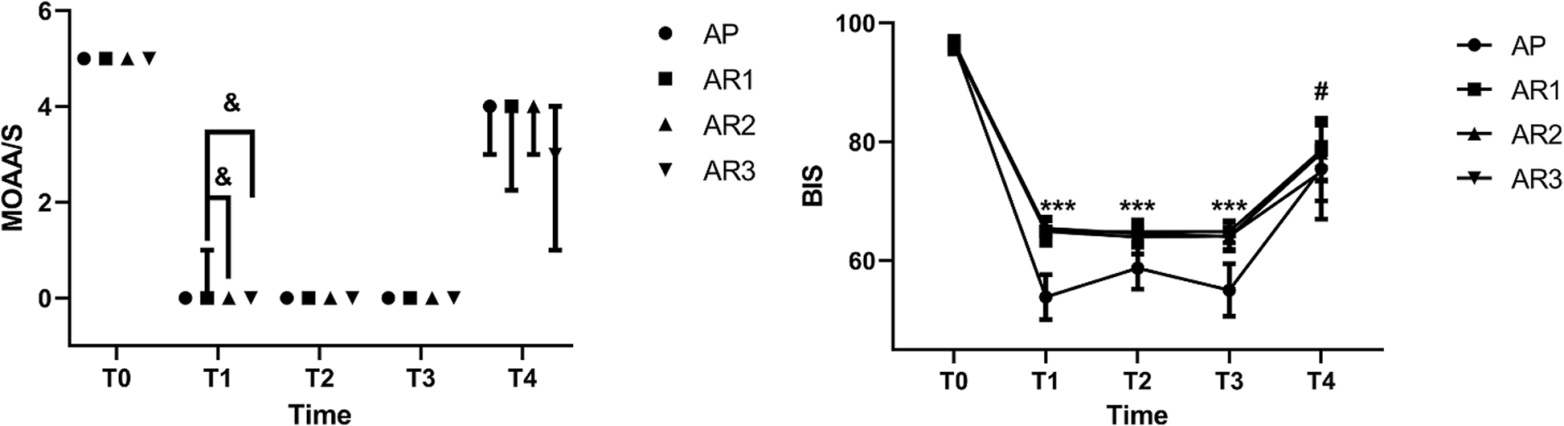
MOAA/S and BIS. Compared with AP group, ***P < 0.001, compared with AR1 group, ^&^ *P* < 0.05, compared with AR3 group, ^#^ *P* < 0.05. MOAA/S was presented as median (25th–75th centiles) and BIS was presented as mean ± SD. Abbreviations: AP alfentanil and propofol; AR1 alfentanil and remimazolam (0.1 mg/kg); AR2 alfentanil and remimazolam (0.15 mg/kg); AR3 alfentanil and remimazolam (0.2 mg/kg)

The MOAA/S scores and vital signs, including HR (*P* = 0.218), SpO_2_ (*P* = 0.327), RR (*P* = 0.208) and BIS (*P* = 0.478), were similar for all study groups at T0 (Supplemental Table 1). The average MAP was significantly higher in AR1-AR3 groups (all *P* < 0.001) than AP group. All doses of remimazolam produce a similar impact on average MAP. (Fig. 4). At T1, compared with the AP group, MOAA/S scores in the AR1 group, AR2 group and AR3 group did not differ significantly. However, the MOAA/S score in the AR1 group was significantly higher compared with AR2 (*P* < 0.05) and AR3 (*P* < 0.05) (Fig. 5), which indicated that patients in the AR2 group and AR3 group had more profound sedation at T1 than those in AR1 group. At T2 and T3, there was no significant difference in MOAA/S scores among all the groups (Fig. 5). BIS values were significantly higher in AR1-AR3 group than AP group (all *P* < 0.05) at T1, T2 and T3 (Table 5, Fig. 5). Nevertheless, there was no statistical difference in BIS values among the remimazolam doses (Table 5, Fig. 5).

**Table 5:** BIS values at T0, T1, T2, T3 and T4

Time point	AP Group (*n* = 27)	AR1 Group (*n* = 28)	AR2 Group (*n* = 30)	AR3 Group (*n* = 29)	*P* value
T0	96.4 ± 1.0	96.2 ± 1.4	96.4 ± 1.1	96.7 ± 1.1	*P* = 0.478
T1	53.9 ± 3.8	65.0 ± 2.7^a^	65.4 ± 2.9^a^	64.9 ± 3.3^a^	*P* < 0.001
T2	58.7 ± 3.5	64.8 ± 3.0^a^	64.6 ± 2.3^a^	64.0 ± 3.7^a^	*P* < 0.001
T3	55.0 ± 4.4	64.9 ± 2.9^a^	64.0 ± 2.4^a^	64.1 ± 2.3^a^	*P* < 0.001
T4	75.4 ± 8.5	78.6 ± 5.0	78.1 ± 4.7	75.0 ± 5.0^b^	*P* = 0.025

*Note*: Data are presented as the mean ± SD

*Abbreviations*: *AP* Alfentanil and propofol, *AR1* Alfentanil followed by remimazolam (0.1 mg/kg), *AR2* Alfentanil followed by remimazolam (0.15 mg/kg), *AR3* Alfentanil followed by remimazolam (0.2 mg/kg)

^a^ *P* < 0.05 vs. AP group

^b^ *P* < 0.05 vs. AR1 group

At T4, more than 50% of patients' MOAA/S scores reached at least 4 in AP, AR1 and AR2 groups and 31.0% in AR3 group. Nevertheless, there were no significant differences among all study groups (Fig. 5). BIS values were statistically lower in the AR3 group than AR1 group (*P* < 0.05, Table 5, Fig. 5). However, compared with the AP group, there was no significant difference in AR1-AR3 groups (Table 5, Fig. 5).

## Discussion

When performing colonoscopy, sedatives and analgesics provide patients comfort, improve examination quality and reduce procedure time [29]. In our study, remimazolam or propofol combined with alfentanil had a 100% successful sedation rate for colonoscopic polypectomy. Moreover, patients who received remimazolam combined with alfentanil had better outcomes.

Propofol is widely used for gastrointestinal endoscopy patients, but it has significant limitations, including individual differences in pharmacokinetics and pharmacodynamics, respiratory depression, hypotension, and lack effective antagonists [30]. Remimazolam is similar to midazolam in pharmacodynamics [31]. A carboxylic ester linkage is introduced into its chemical structure, which acts similarly to remifentanil and can be metabolized by nonspecific tissue esterases in blood. A study indicated that remimazolam achieved a moderate depth and duration of sedation by titration and could maintain the stability of vital signs even in deep sedation [32] and was quickly reversed by flumazenil (median time to alert entirely was 1.0 min) [33]. In our research, we observed that patients in remimazolam groups took less time to be fully alert than propofol group. The time to full alert in remimazolam groups was in the range of 3–10 min, which was similar to the findings in previous studies [20, 28]. Patients also reached the standard of leaving PACU more quickly in remimazolam groups than propofol group. However, Chen et al. found no significant difference in the time to full alert between remimazolam and propofol in their phase III clinical trial [21]. Except for the differences in clinical practice (colonoscopy vs. colonoscopic polypectomy), The use of anesthetics also varies (fentanyl-remimazolam vs. alfentanil-remimazolam). These aspects all had impacts on the time to full alert. Also, Tian et al. indicated that elderly patients who received 0.2 mg/kg remimazolam took significantly longer to recover than propofol in upper gastrointestinal endoscopy [34]. Besides the age of patients, differences in the administration of remimazolam and the dosage of propofol may lead to the opposite result.

Intraoperative hypotension is associated with organ injury and poor outcomes [35]. An exploratory analysis suggested more episodes of hypotension when propofol was used for sedation than remimazolam [36]. Furthermore, a series of studies have shown that remimazolam had more stable hemodynamics and a lower incidence of hypotension in multiple surgical types [34, 37–41]. In our study, the incidence of hypotension was lower and a higher average MAP existed in AR groups than AP group. As for the average MAP and the incidence of hypotension among the AR1, AR2 and AR3 groups, no difference was found.

According to the package insert of remimazolam in our study recommends that administer an initial dose intravenously as a 7 mg push injection over a 1-min time period. If necessary, administer supplemental doses of 2.5 mg intravenously over a 15-s time period. We used the mode of single administration in three doses of remimazolam groups. The metabolism of remimazolam was dose-independent and followed the first-order kinetic model [42]. We found that patients received the first additional dose at inconsistent intervals after sedation induction. Patients in the AR2 and AR3 groups took more extended intervals. However, the subsequent addition of remimazolam was performed at an interval of 5–8 min for all remimazolam groups, which was consistent with the context-sensitive half time of remimazolam [43]. Besides, the duration of surgery among the groups did not differ significantly. Therefore, there was no difference in the total doses of remimazolam among the AR1-AR3 groups.

It was highlighted that there were some statistically different results among the AR1, AR2 and AR3 groups. First, the MOAA/S score in the AR1 group was higher than AR2 and AR3 groups at T1, indicating that 0.15 mg/kg and 0.2 mg/kg remimazolam could achieve better sedation at the beginning of the operation. It was necessary for colonoscopic polypectomy because colonoscope insertion, especially in the sigmoid colon, was time-consuming and technically challenging [44], so patients in the initial stage were insulted more severely. Second, BIS values in AR1 group were significantly higher than AR3 group at T4, and the time to full alert was a little longer in AR3 group compared with the AR1 and AR2 groups. Based on the analysis above, although there was no significant difference in the total doses of remimazolam and alfentanil, and the rate of side effects was also similar among the AR1-AR3 groups, remimazolam 0.15 mg/kg might be the first choice for sedation induction because of the suitable depth of sedation at the beginning of the operation and the advantage of a faster recovery in relatively shorter surgery. This result needs to be confirmed by a large sample study further.

BIS monitoring was used to assess the appropriate sedation levels of anesthesia. We found that BIS values (58–69) were significantly higher in AR1-AR3 groups compared with AP group (45–63), which was consistent with previous studies [45–48]. None of the patients reported intraoperative awareness during surgery for all study groups. We also observed that BIS values were above 60 in some patients even though they received 0.2 mg/kg remimazolam for sedation induction. The BIS values among AR1, AR2 and AR3 groups showed no statistical difference. Therefore, appropriate ranges of the BIS for remimazolam sedation still need more research [47].

In our study, it should also be noted that although remimazolam and propofol can produce enough sedation for colonoscopic polypectomy, propofol is more expensive than remimazolam. In our institution, the cost of a 25 mg vial of remimazolam besylate is 69.8¥, and a 500 mg vial of propofol is 178.3¥. Patients in AR groups needed at most two vials of remimazolam in our research. Based on the suitable sedation and the lower cost, we prefer remimazolam combined with alfentanil sedation for colonoscopic polypectomy.

This study has the following limitations: First, it was a single-center study and the sample size is limited. Hence, our findings did not possess universality. Second, remimazolam should be given additional doses several times due to the 2020 edition package insert in this study. So continuous transfusion of remimazolam could be considered for subsequent studies with the update of the instructions. Third, we only conducted a 24-h postoperative follow-up to evaluate postoperative adverse reactions. Long-term complications were not assessed. Further studies are needed to validate the present findings.

## Conclusion

In conclusion, remimazolam combined with alfentanil was a safe alternative for sedation and analgesia during the colonoscopic polypectomy. In particular, there was a low incidence of hypotension and fast full alert. As a novel intravenous benzodiazepine, the safety and feasibility of the anesthesia scheme of remimazolam should be further evaluated.

## Supplementary Information

**Additional file 1. Supplemental Table 1. **Vital signs at T0, T1, T2, T3 and T4; Data are presented as mean ± SD and median (25th–75th centiles). We summarized and analyzed vital signs of patients at T0, T1, T2, T3 and T4.

## Data Availability

All data generated or analyzed during this study are included in this published article and its supplementary information files.

## Abbreviations

ANOVA: Analysis of variance
AP: Alfentanil (10 μg/kg) and propofol (2 mg/kg)
AR1: Alfentanil (10 μg/kg) followed by remimazolam (0.1 mg/kg)
AR2: Alfentanil (10 μg/kg) followed by remimazolam (0.15 mg/kg)
AR3: Alfentanil (10 μg/kg) followed by remimazolam (0.2 mg/kg)
ASA: American Society of Anesthesiologists
BIS: Bispectral index; BMI: body mass index
BP: Blood pressure
CONSORT: Consolidated Standards of Reporting Trials
CRF: Case report form
GABA_A_: γ-Aminobutyric acid subtype A
HR: Heart rate
IV: Intravenous
MAC: Monitored anesthesia care
MAP: Mean arterial pressure
MOAA/S: Modified Observers Assessment of Alertness and Sedation
NA: Not applicable
PACU: Post anesthesia care unit
PASS: Power Analysis and Sample Size
RR: Respiratory rate
SD: Standard deviation
SPSS: Statistical Package for Social Sciences
SpO_2_: Oxygen saturation
