# Supplementary material for: Sedative effect of remimazolam combined with alfentanil in colonoscopic polypectomy: a prospective, randomized, controlled clinical trial
Source: BMC Anesthesiol. 2022 Aug 16;22:262. doi: 10.1186/s12871-022-01805-3 (PMC9380378; doi:10.1186/s12871-022-01805-3)
Supplement: Supplementary file 1 — Additional file 1. Supplemental Table 1. Vital signs at T0, T1, T2, T3 and T4; Data are presented as mean ± SD and median (25th–75th centiles). We summarized and analyzed vital signs of patients at T0, T1, T2, T3 and T4. [file 12871_2022_1805_MOESM1_ESM.docx]

**Supplementary Information**

**Additional file 1: Supplemental Table 1.** Vital signs at T0, T1, T2, T3 and T4; Data are presented as mean ± SD and median (25th–75th centiles). We summarized and analyzed vital signs of patients at T0, T1, T2, T3 and T4.

AP alfentanil (10μg/kg) and propofol (2mg/kg); AR1 alfentanil (10μg/kg) followed by remimazolam (0.1 mg/kg); AR2 alfentanil (10μg/kg) followed by remimazolam (0.15 mg/kg); AR3 alfentanil (10μg/kg) followed by remimazolam (0.2 mg/kg); HR heart rate; NA not applicable; RR respiratory rate; SD standard deviation; SpO_2_ oxygen saturation. P values represented one-way ANOVA, Welch ANOVA and Kruskal-Walli’s test.

| **T0** | AP Group  (n=27) | AR1 Group  (n=28) | AR2 Group  (n=30) | AR3 Group  (n=29) | *P* value |
| --- | --- | --- | --- | --- | --- |
| BIS | 96.4±1.0 | 96.2±1.4 | 96.4±1.1 | 96.7±1.1 | *P* =0.478 |
| SpO_2_, % | 100% (99%, 100%) | 100% (99%, 100%) | 100% (100%, 100%) | 100% (100%, 100%) | *P* =0.327 |
| RR, bpm | 18.0 (16.0, 18.0) | 17.0 (15.0, 18.0) | 17.0 (15.0, 18.0) | 16.0 (15.0, 18.0) | *P* =0.208 |
| HR, bpm | 76.85±13.01 | 77.29±11.47 | 81.67±12.53 | 81.76±9.51 | *P* =0.218 |
| **T1** | | | | | |
| SpO_2_, % | 100% (99%, 100%) | 100% (99%, 100%) | 100% (99%, 100%) | 100% (99%, 100%) | *P* =0.885 |
| RR, bpm | 12.00 (12.00, 13.00) | 12.00 (11.00, 13.00) | 12.00 (11.00, 13.25) | 13.00 (11.00, 13.50) | *P* =0.892 |
| HR, bpm | 68.70±12.59 | 72.00±9.92 | 74.73±11.15 | 72.45±9.25 | *P* =0.218 |
| **T2** | | | | | |
| SpO_2_, % | 100% (99%, 100%) | 100% (99%, 100%) | 100% (99%, 100%) | 100% (99%, 100%) | *P* =0.928 |
| RR, bpm | 13.00 (11.00, 14.00) | 13.00 (12.00, 14.00) | 12.00 (11.00, 13.00) | 13.00 (12.00, 14.00) | *P* =0.150 |
| HR, bpm | 70.04±12.14 | 71.93±9.21 | 73.77±10.55 | 71.62±9.38 | *P* =0.602 |
| **T3** | | | | | |
| SpO_2_, % | 100% (99%, 100%) | 100% (99%, 100%) | 100% (99%, 100%) | 100% (99.5%, 100%) | *P* =0.824 |
| RR, bpm | 12.00 (12.00, 12.00) | 12.00 (11.25 12.00) | 12.00 (11.00, 13.00) | 12.00 (12.00, 13.00) | *P* =0.266 |
| HR, bpm | 71.78±11.33 | 71.25±9.36 | 74.53±10.92 | 71.41±9.65 | *P* =0.581 |
| **T4** | | | | | |
| SpO_2_, % | 100% (99%, 100%) | 100% (99%, 100%) | 100% (99%, 100%) | 100% (99%, 100%) | *P*=0.866 |
| RR, bpm | 14.00 (13.00, 15.00) | 14.00 (13.00, 15.00) | 14.50 (14.00, 15.00) | 14.00 (14.00, 15.00) | *P*=0.689 |
| HR, bpm | 75.30±13.79 | 75.39±11.06 | 78.10±10.13 | 75.55±10.86 | *P*=0.750 |
